# An environment-guided visual–temporal deep learning framework for early disease detection in greenhouse horticultural crops

**DOI:** 10.3389/fpls.2026.1796407

**Published:** 2026-04-07

**Authors:** Jiayu Xiang, Jinrui Ge, Ceteng Fu, Jiayi Huang, Mingkun Lu, Zhonghao Zhang, Yihong Song, Shuo Yan

**Affiliations:** 1 China Agricultural University, Beijing, China; 2 Peking University, Beijing, China

**Keywords:** computer vision for plant science, early disease detection, environmental sensor data, greenhouse horticulture, precision plant protection

## Abstract

**Introduction:**

In protected horticultural production, early disease identification and precise intervention are critical for safeguarding crop yield and quality while reducing chemical pesticide inputs. However, early-stage greenhouse diseases often exhibit extremely subtle visual symptoms, and their occurrence and progression are highly dependent on environmental condition variations, making stable and reliable early warning difficult to achieve using conventional methods based on single visual information or simple multimodal fusion.

**Methods:**

To address this challenge, a visual–environment joint early disease perception framework for greenhouse horticultural crops is proposed. Through an environment-guided visual attention mechanism and a spatial–temporal joint modeling strategy, environmental variables such as temperature, humidity, vapor pressure deficit, and CO_2_ concentration are transformed from passive features into active priors, thereby guiding visual feature learning and enhancing sensitivity to weak disease signals. The proposed method is systematically validated on a real-world greenhouse multimodal temporal dataset.

**Results:**

Experimental results demonstrate that the proposed approach achieves an accuracy of 91.3%, a recall of 88.9%, and an F1-score of 89.8% in overall disease detection tasks, significantly outperforming multiple baseline models based on convolutional neural networks (CNNs), Transformers, and existing multimodal fusion strategies. In early-stage disease detection scenarios, early precision and early recall reach 88.5% and 86.1%, respectively, with the lead time extended to 2.7 days, indicating a clear advantage in early warning capability. Ablation studies further verify the critical roles of environment-guided attention, spatial–temporal joint modeling, and the joint loss function in improving early detection performance and stability.

**Discussion:**

This study provides a practically valuable technical pathway for early intelligent warning and precise regulation of greenhouse crop diseases. By integrating environmental dynamics with visual perception, the proposed framework improves the sensitivity and robustness of early disease detection in complex greenhouse conditions, showing strong potential for practical deployment in protected horticulture.

## 1 Introduction

In greenhouse horticulture, diseases and pests are critical biotic stressors limiting yield and quality improvement Fanourakis et al. (2025). The controllable nature of greenhouses allows for precise environmental regulation if early detection and risk warnings are achieved, which can suppress disease spread, reduce pesticide use, and enhance production safety Maraveas (2022). Thus, early detection technologies are fundamental to intelligent greenhouse management and green, efficient agriculture Venkatesh et al. (2024). However, at the initial stage, disease symptoms are often extremely weak, with small lesions, low contrast, and unstable spatial features, posing significant challenges to automated recognition Khan et al. (2025).

Traditional detection has relied on manual inspection or rule-based image processing and machine learning Hong (2025). While manual identification leverages expertise, it is inefficient, subjective, and unsuitable for large-scale greenhouse production Jin et al. (2024). Early automated methods typically used handcrafted features and classifiers like support vector machine (SVMs) Zhang et al. (2022b), but they are sensitive to illumination, cultivar differences, and complex backgrounds Singh et al. (2025). Moreover, they struggle to extract discriminative features from early, subtle symptoms and often ignore the dynamic disease progression, failing to meet early-warning needs Dolatabadian et al. (2025).

Recently, deep learning, especially convolutional neural networks (CNNs) and transformers, has advanced agricultural disease recognition Zeng et al. (2025); Zhang et al. (2021a). Deep models can learn high-level features end-to-end, outperforming traditional methods in visible lesion recognition Upadhyay et al. (2025). Attention mechanisms and multi-scale fusion further improve handling of complex backgrounds and fine-grained symptoms Qian et al. (2025). However, most deep learning approaches still rely solely on RGB images and explicit visual symptoms Nahian (2025). When diseases are latent, visual cues are extremely limited, making stable early detection difficult with vision-only data Kim et al. (2023). On the other hand, greenhouse environmental sensors continuously collect variables like temperature, humidity, and CO_2_, which are closely linked to disease occurrence Bicamumakuba et al. (2025). Specific environmental conditions and fluctuation patterns often favor pathogen spread Kumar and Mukhopadhyay (2025). However, in most multimodal recognition studies, environmental data are merely auxiliary features fused simply with visual data, without sufficiently modeling the spatiotemporal coupling between environmental variations and symptom development Ross et al. (2022). Inconsistencies in data sampling and resolution complicate alignment, and environmental data may contain irrelevant noise that can interfere with visual feature learning Zhou et al. (2025). Moreover, disease progression is a temporal process where environmental changes often precede visible symptoms Das et al. (2022). Most methods use static or short-term modeling, failing to mine long-term associations between environmental trends and disease evolution or explicitly model spatial lesion diffusion Yang et al. (2023). Therefore, achieving deep collaborative modeling of visual and environmental temporal information within a unified framework, while using environmental cues to guide visual learning, remains a critical challenge Farooq et al. (2022). Recent studies have explored multimodal fusion, transformer-based frameworks, multispectral monitoring, and cross-domain generalization to improve early detection and recognition Zhang et al. (2022a); Lu et al. (2024); Khan et al. (2024); Li et al. (2025b).

Based on the above analysis, a visual–environmental joint perception method for early disease detection in greenhouse horticultural crops is proposed. A dual-branch multimodal deep learning framework is constructed to achieve temporal alignment and collaborative modeling of rgb image sequences and multidimensional environmental sensor data. In this design, environmental information is no longer treated as a passive auxiliary input, but is explicitly leveraged to dynamically modulate the spatial response distribution of visual features through an environment-guided attention mechanism, thereby enhancing the model sensitivity to weak early-stage disease characteristics. Meanwhile, a spatial attention mechanism is introduced to characterize potential lesion diffusion regions, and a temporal attention mechanism is combined to model long-term dependencies between environmental variation trends and disease progression, enabling joint spatial–temporal modeling of the disease evolution process.

The main contributions of this study are summarized as follows:

1. An environment-guided visual attention modeling strategy is proposed, in which greenhouse environmental factors are explicitly incorporated as prior information into the visual feature learning process, effectively alleviating the challenge of weak and insufficiently discriminative visual features in early disease stages.
2. A joint spatial–temporal attention mechanism is constructed to simultaneously characterize spatial lesion diffusion patterns and temporal disease evolution driven by environmental changes within a unified framework, thereby enhancing the capability for modeling dynamic disease processes.
3. A multimodal temporal disease dataset is constructed and validated under real greenhouse conditions. Unlike existing public datasets that predominantly rely on static, single-modality images capturing explicit late-stage symptoms in controlled environments, the proposed dataset emphasizes the continuous dynamic evolution of early weak symptoms strictly aligned with multi-dimensional environmental sensor data. Extensive comparative experiments and ablation analyses are conducted on this dataset to comprehensively demonstrate the effectiveness and robustness of the proposed method for early disease detection tasks.

## 2 Related work

### 2.1 Visual-based disease recognition methods for horticultural crops

Visual-based disease recognition for horticultural crops is a key element of intelligent agricultural perception, aiming to distinguish health status by analyzing color, texture, and morphological changes on plant surfaces Qadri et al. (2024). Early research primarily utilized traditional computer vision and machine learning, relying on manually designed features and classifiers like SVMs, k-Nearest Neighbor (k-NN), or random forests Domingues et al. (2022); Hayit et al. (2024). However, these methods were sensitive to environmental variations, cultivar differences, and diverse symptom expressions, limiting their robustness Zsögön et al. (2022). The advent of deep learning, particularly CNNs, has significantly advanced the field by enabling automatic feature learning, leading to superior performance in classification and localization tasks over traditional methods Petchiammal et al. (2023); Nawaz et al. (2022). Models based on architectures like AlexNet, VGG, and ResNet, often enhanced with multi-scale fusion or attention mechanisms, have achieved high accuracy for distinct symptoms Thakur et al. (2023); Lin et al. (2022). Nevertheless, their effectiveness heavily depends on clearly visible lesions and struggles with early-stage symptoms characterized by subtle visual changes in single RGB images Reza et al. (2025). Recently, Transformer models have been introduced for their global modeling capacity via self-attention Zhang et al. (2021b), showing promising results in handling complex backgrounds compared to CNNs Chitta et al. (2024); Clement (2025); Mehdipour et al. (2025). Yet, as they also primarily process static images, their performance remains constrained by the explicitness of visual features and is often insufficient for reliably detecting early disease states before stable lesion structures form Khan et al. (2022); Kong et al. (2022).

### 2.2 Applications of multimodal sensors in greenhouse crop monitoring

The highly controllable greenhouse environment provides a foundation for deploying multimodal sensors, where factors like temperature, humidity, and CO_2_ concentration critically influence disease mechanisms. Consequently, environmental sensor data are increasingly utilized for crop monitoring and disease risk analysis, typically employing statistical or machine learning models to uncover variable-disease associations for outbreak prediction Yang et al. (2025); Mujtaba et al. (2025); Ramani et al. (2025); Lee and Yun (2023). In multimodal perception, fusing environmental data with visual information aims to enhance recognition robustness by leveraging modality complementarity. However, most current methods employ shallow strategies like feature concatenation or decision-level fusion, failing to adequately model the intrinsic causal and temporal relationships between environmental cues and visual symptoms Li et al. (2023); Shovon et al. (2023); Liu and Wang (2024); Lou et al. (2024); Liu et al. (2024). Furthermore, effective fusion is complicated by discrepancies in sampling frequency and resolution between continuous environmental data and sparse image acquisition. Without proper temporal alignment, environmental inputs may introduce noise. More critically, the influence of environmental variables is often stage- and condition-specific, yet existing approaches generally lack fine-grained modeling of this dynamic role, treating environmental data as a passive feature source rather than an active guide for visual learning Barbedo (2022); Bhujade et al. (2024); Vasamsetti et al. (2025); Ju et al. (2023). Therefore, establishing a deep, principled collaboration where environmental data provide active prior constraints, particularly during early disease stages, remains a key challenge in multimodal greenhouse disease monitoring.

### 2.3 Progress in temporal modeling and attention mechanisms for agricultural scenarios

Agricultural production exhibits strong temporal continuity, as crop growth, environmental variation, and disease development are inherently dynamic processes. Consequently, temporal modeling methods are widely applied in agricultural scenarios. Traditional approaches, primarily based on statistical models or recurrent neural networks (RNNs), model historical observations to predict future trends. With the advancement of deep learning, long short-term memory (LSTM) networks and gated recurrent units (GRUs), capable of capturing long-term dependencies, have been extensively used for tasks like environmental forecasting, growth monitoring, and disease occurrence prediction Zhou et al. (2022); Kumar et al. (2024); Wang and Li (2025); Lin (2024). Building on this, attention mechanisms have become a key tool for enhancing temporal modeling. By assigning dynamic weights to different time steps, they allow models to focus on the most relevant historical information, mitigating information decay in long sequences. Recent years have seen the introduction of attention-based methods into agricultural time series analysis to identify key stages or anomalous patterns in environmental variation, demonstrating potential for disease early warning Cheng et al. (2022); Zhu et al. (2024); Saini et al. (2022). However, most existing research applies temporal attention to single-modality sequences, with limited exploration of its joint modeling with spatial information. In disease detection, the spatial distribution and diffusion of lesions are crucial, especially in early stages when spread is localized. Pure temporal modeling cannot reflect spatial evolution, while sole reliance on spatial attention fails to capture the cumulative influence of environmental changes. Thus, integrating spatial and temporal attention within a unified framework for comprehensive disease evolution modeling remains an underexplored research area Li et al. (2025a); Salamai et al. (2023); Zhao et al. (2023); Zhu et al. (2024).

## 3 Materials and method

### 3.1 Data collection

**Figure 1:**
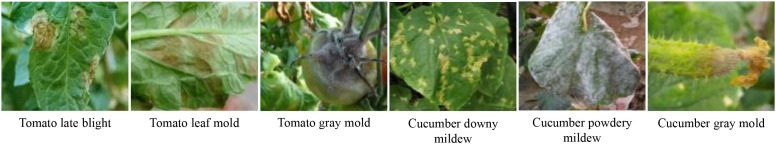
Instances of greenhouse diseases within the dataset.

The multimodal disease dataset used in this study was collected in a modern facility agriculture greenhouse located in Wuyuan county, Bayannur city, Inner Mongolia, China, as shown in **Table 1**. This region exhibits typical temperate continental climate characteristics, with pronounced diurnal temperature variation. During greenhouse production, ventilation, heating, and irrigation are frequently regulated, which facilitates the formation of realistic application scenarios involving disease occurrence and evolution. The target crops for data collection were two representative greenhouse horticultural crops, namely tomato and cucumber. The collection period spanned from March 2024 to August 2024, covering key growth stages including vegetative growth, flowering and fruit-setting, as well as periods with high disease incidence. Particular attention was paid to common and representative greenhouse diseases during the acquisition process. For tomato, the main disease types included tomato late blight, tomato leaf mold, and tomato gray mold, while cucumber diseases mainly consisted of cucumber downy mildew, cucumber powdery mildew, and cucumber gray mold, as showed in **Figure 1**. In addition, healthy plant samples were retained to construct a complete disease occurrence spectrum. Although the dataset encompasses various specific diseases across two crops, the annotation and subsequent modeling framework deliberately construct a unified stage-based classifier (Healthy, Early, Symptomatic) rather than a fine-grained disease-specific classifier. This approach is grounded in three practical considerations. First, during the early incubation phase, initial visual symptoms (e.g., slight chlorosis and minor texture disturbances) are highly generic and visually indistinguishable across different pathogens, making fine-grained classification on such weak features highly error-prone. Second, the targeted fungal and oomycete diseases share heavily overlapping environmental triggers, primarily driven by high humidity and specific temperature fluctuations. Third, from a practical greenhouse management perspective, the immediate intervention triggered by an early warning typically involves broad-spectrum environmental regulation (e.g., enhancing ventilation, adjusting humidity) rather than disease-specific chemical spraying, which is usually reserved for the symptomatic stage. Thus, a unified early risk perception model directly aligns with the operational workflows of proactive environmental control.

**Table 1:** Composition and scale statistics of the greenhouse multimodal crop disease dataset.

Data modality	Sampling setting	Data source	Data volume
RGB images (tomato)	2–4 acquisitions per day	Fixed/mobile cameras	10,240 images
RGB images (cucumber)	2–4 acquisitions per day	Fixed/mobile cameras	8,180 images
Air temperature	1 min continuous sampling	Greenhouse environmental sensors	320,000 records
Relative humidity	1 min continuous sampling	Greenhouse environmental sensors	320,000 records
Vapor pressure deficit	1 min continuous sampling	Computed from sensors	320,000 records
CO_2_ concentration	1 min continuous sampling	Greenhouse environmental sensors	320,000 records
Disease stage (healthy)	Event-driven annotation	Expert plant protection labeling	6,140 records
Disease stage (early)	Event-driven annotation	Expert plant protection labeling	6,120 records
Disease stage (symptomatic)	Event-driven annotation	Expert plant protection labeling	6,160 records

Visual data were primarily obtained using fixed high-resolution industrial cameras deployed inside the greenhouse and manually assisted mobile acquisition devices. The fixed cameras were installed approximately 1.2–1.5 m above the crop canopy and configured with an approximately vertical top-down view to periodically capture leaf and fruit regions, enabling continuous recording of subtle appearance changes before and after disease onset. The mobile acquisition component was conducted by operators at different time periods to obtain close-range supplementary images of selected plants, with particular emphasis on weak symptoms during disease incubation and early stages that are difficult to distinguish by naked eye observation, such as slight leaf chlorosis, initial spot formation, and minor texture disturbances. To ensure rigorous annotation, the “early stage” category was strictly defined based on both morphological and retrospective temporal criteria. Morphologically, early-stage samples exhibit disease-related visual anomalies covering less than 5% of the observable leaf or fruit surface area, without evident tissue necrosis, structural collapse, or sporulation. Temporally, these labels were retrospectively validated by plant protection experts through continuous tracking; a sample was conclusively categorized as “early stage” only if the target plant developed typical, symptomatic disease features within the subsequent 1 to 3 days. This retrospective confirmation effectively distinguishes true disease incubation from transient physiological stress or environmental artifacts. Environmental data were continuously collected using a multi-source sensor network deployed within the greenhouse. Sensor nodes were evenly distributed according to crop row spacing and planting layout, and environmental conditions were synchronously monitored at different heights to capture both near-canopy and ambient air states. The collected environmental variables included air temperature, relative humidity, vapor pressure deficit, and CO_2_ concentration, where temperature, relative humidity, and CO_2_ concentration were directly measured by digital sensors, and vapor pressure deficit was calculated based on temperature and relative humidity measurements.

### 3.2 Data preprocessing and augmentation strategy

In early disease detection tasks for greenhouse horticultural crops, substantial discrepancies among multimodal data in terms of temporal scale, sampling frequency, and noise characteristics introduce fundamental challenges for subsequent joint modeling. RGB images are typically acquired at discrete time points, with sampling frequency constrained by camera deployment and storage conditions, whereas environmental sensor data are continuously recorded at minute- or even second-level resolution to capture microclimate variations within the greenhouse. Without appropriate temporal alignment and noise suppression, latent disease-inducing signals embedded in environmental information are difficult to be effectively associated with visual symptoms and may even introduce additional interference, thereby weakening the model capability to perceive weak early-stage disease features. Therefore, systematic preprocessing and augmentation of both environmental and visual data prior to multimodal joint modeling are essential steps for ensuring model stability and generalization performance.

From a methodological perspective, the core objective of multimodal temporal alignment is to map data streams with heterogeneous sampling frequencies into a unified temporal reference framework, such that environmental features indexed at the same time point can faithfully reflect the actual growth stage and disease risk corresponding to each image. Let the raw environmental sensor time series be denoted as $E={\left\{(e_{i},t_{i})\right\}}_{i=1}^{N}$, where $e_{i}\in {\mathbb{R}}^{d}$ (with *d* = 4 in this study) represents a *d*-dimensional environmental variable vector collected at time *t_i_*, consisting of temperature, relative humidity, vapor pressure deficit (VPD), and CO_2_ concentration. Meanwhile, the RGB image sequence is denoted as $I={\left\{(I_{j},\tau_{j})\right\}}_{j=1}^{M}$, where I*_j_* is the image frame captured at time *τ_j_*. As *N* ≫ *M* generally holds and *t_i_* and *τ_j_* are not strictly aligned, resampling and aggregation of the environmental sequence are required to obtain environmental representations corresponding one-to-one with image timestamps.

A sliding time-window-based aggregation strategy is adopted for environmental data. For each image I*_j_*, a temporal window of length 2Δ*t* (with Δ*t* empirically set to 30 minutes in this study) is constructed centered at its acquisition time *τ_j_*, defined as 𝒲*_j_* = [*τ_j_* − Δ*t,τ_j_* + Δ*t*]. Environmental observations within this window are statistically aggregated to obtain the corresponding environmental feature vector ${\tilde{e}}_{j}$, which can be expressed as Equation 1.

(1) $${\tilde{e}}_{j}=\frac{1}{\left|W_{j}\right|}\sum_{t_{i}\in W_{j}}e_{i},$$

where $\left|W_{j}\right|$ denotes the number of environmental observations within the window. This aggregation scheme preserves the overall trend of environmental variation while effectively suppressing random noise induced by instantaneous fluctuations, thereby yielding environmental features that are more consistent with the temporal characteristics of disease incubation and development. In practical field settings, acquiring the complete environmental sequence within [*τ_j_* − Δ*t,τ_j_* + Δ*t*] inherently introduces a bounded observation delay of Δ*t* (30 minutes in our implementation) before a prediction for the image at *τ_j_* can be executed. However, given that greenhouse crop diseases evolve progressively on a macro-temporal scale of days (as evidenced by our average lead time of 2.7 days), this brief buffering interval is completely negligible for timely agricultural interventions. In modern greenhouse management, climate control systems (e.g., ventilation, dehumidification, and irrigation) and manual inspections typically operate on hourly or diurnal periodic schedules rather than requiring sub-second instantaneous responses. Therefore, executing the disease perception framework with a short temporal buffer perfectly aligns with real-world agricultural workflows, ensuring robust environmental noise suppression without compromising the practical utility of early disease warning.

During environmental data preprocessing, anomalies and noise disturbances cannot be ignored. Long-term sensor operation may be affected by hardware drift, communication latency, or short-term interference, leading to abrupt changes in environmental variables. Directly feeding raw data into the model may result in feature distribution shifts and interfere with the learning of disease-related patterns. To mitigate this issue, temporal smoothing is applied to environmental time series to enhance continuity. Specifically, for each environmental variable dimension *e*^(^*^k^*^)^(*t*), an exponentially weighted smoothing strategy is employed, formulated as Equation 2.

(2) $${\hat{e}}^{\left(k\right)}(t)=\alpha e^{\left(k\right)}(t)+(1-\alpha ){\hat{e}}^{\left(k\right)}(t-1),$$

where *α* ∈ (0,1) is the smoothing coefficient (set to 0.2 in our experiments) that balances the contribution of the current observation and historical trends. It allows high-frequency noise to be effectively attenuated while retaining slow-varying trends associated with disease development.

After temporal alignment and smoothing, normalization is further applied to environmental features to prevent numerical scale discrepancies among variables from adversely affecting model training. Let the mean and standard deviation of a given environmental variable over the training set be *µ_k_* and *σ_k_*, respectively. The normalized form is defined as Equation 3.

(3) $$e_{\text{norm}}^{\left(k\right)}=\frac{e^{\left(k\right)}-\mu_{k}}{\sigma_{k}}.$$

This operation facilitates faster model convergence and prevents any single environmental factor with a large numerical magnitude from dominating the multimodal fusion process, thereby ensuring a balanced contribution of different environmental variables.

For visual data, considering the characteristics of early disease detection, where symptoms are weak and structural patterns are unstable, the design of data augmentation strategies follows the principle of minimal perturbation while preserving semantic integrity. Unlike explicit lesion recognition tasks that commonly employ large rotations or strong color distortions, excessive image transformations may destroy the subtle texture or color variations that constitute the only visual cues of early disease stages, leading the model to learn spurious features unrelated to disease progression. Therefore, lightweight image augmentation strategies are adopted to enhance sample diversity while preserving the authenticity of original disease structures.

Specifically, let the original image be denoted as I, and the augmented image I^′^ is generated through a set of controlled transformation functions 𝒯 (·), expressed as Equation 4.

(4) $$I^{\prime }=T(I;\theta ),$$

where *θ* represents the augmentation parameter set, including mild brightness perturbation (randomly adjusted within ±10%), slight contrast adjustment (randomly adjusted within ±10%), and small random cropping (preserving at least 95% of the original image area). These operations mainly simulate natural variations in greenhouse illumination conditions and minor shifts in imaging viewpoints, without introducing strong structural changes inconsistent with disease development processes.

The aforementioned visual enhancement strategy exposes the model to more samples with similar semantics but slightly different appearances, enhancing robust perception of early, weak disease features. At the same time, by strictly controlling the enhancement intensity, the fine-grained texture and local color differences of early lesions are preserved, avoiding information loss.

### 3.3 Proposed method

#### 3.3.1 Overall

**Figure 2:**
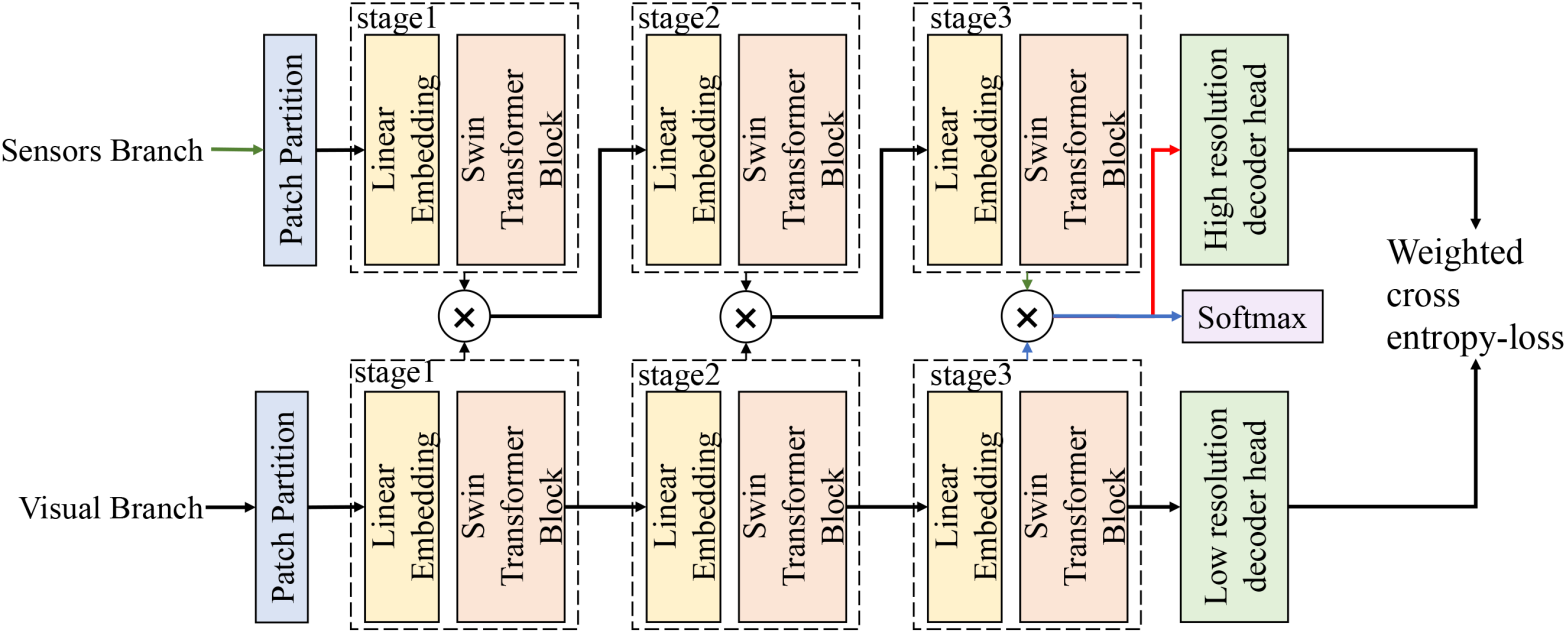
Overall framework illustration showing the dual-branch architecture. The Sensors Branch and Visual Branch process environmental data and RGB image patches in parallel through a hierarchical Swin Transformer backbone (Stages 1 to 3) following an initial Patch Partition. At each stage, an environment-guided cross-attention mechanism (⊗) fuses the multi-scale representations, actively utilizing environmental semantics to guide visual feature learning. The multi-scale fused features are then processed by high-resolution and low-resolution decoder heads, with predictions optimized end-to-end via a weighted cross-entropy loss.

A visual–environment dual-branch joint perception framework is constructed from the perspective of model design, as shown in **Figure 2**, where the inputs consist of an aligned image sequence {I*_j_*} and the corresponding aligned environmental vector sequence $\left\{{\tilde{e}}_{j}\right\}$, including temperature, humidity, VPD, and CO_2_. Both modalities are synchronously fed into the network under the same temporal index *j*. The upper branch corresponds to the sensor branch, in which ${\tilde{e}}_{j}$ is first projected through a linear mapping to obtain an environmental embedding **s***_j_*, and a sequence of environmental tokens is formed along the temporal dimension. Through multi-level hierarchical encoding, progressively more semantically expressive environmental representations $\left\{S_{j}^{\left(l\right)}\right\}$ are generated and serve as risk-aware priors for subsequent cross-modal interactions. The lower branch corresponds to the image branch, where I*_j_* is partitioned into patches and linearly embedded into visual tokens, which are then processed by a hierarchical Swin Transformer encoder. Through stages 1–4 with window-based self-attention and patch merging, multi-scale visual features $\left\{V_{j}^{\left(l\right)}\right\}$ are obtained while preserving fine-grained spatial details for subsequent localization of early weak symptoms. Critical cross-modal interactions are performed after each backbone stage through the environment-guided visual attention module. This module bridges the two branches by utilizing the environmental embeddings **s***_j_* to generate conditional regulatory factors. These factors dynamically modulate the multi-scale visual features $\left\{V_{j}^{\left(l\right)}\right\}$ channel-wise, effectively using environmental risk priors to amplify subtle visual responses in potential disease regions, yielding the modulated representations ${\tilde{F}}_{j}^{\left(l\right)}$. Subsequently, these modulated features are fed into the spatial–temporal joint attention module. This module builds directly upon the environment-guided outputs, first applying spatial attention to capture local lesion diffusion patterns, and then aggregating these spatially enhanced features across the temporal sequence using bidirectional temporal attention to model the long-term disease evolution process. The fused outputs are injected back into the backbone feature stream via residual connections to prevent excessive overwriting of original visual evidence. After the hierarchical interactions and spatial–temporal modeling are completed, the final-stage outputs are combined with high-level visual semantics and fed into the prediction head to produce disease region or stage outputs ${\hat{y}}_{j}$. Auxiliary outputs can be introduced at intermediate scales to enable deep supervision, stabilizing gradients during early-stage training and strengthening the learning of weak symptoms. The entire network is optimized end-to-end, with a weighted cross-entropy loss as the main objective and joint constraints applied across multiple scales, thereby forming a complete processing pipeline driven by environmental priors, cross-attention fusion, and spatial semantic localization. To explicitly clarify the data flow of the final integrated model, the input and output variables are formally defined for both the training and testing stages. During the training stage, the model inputs consist of the aligned image sequence {I*_j_*}, the corresponding environmental vector sequence { ${\tilde{e}}_{j}$} (comprising temperature, relative humidity, VPD, and CO_2_), and the ground-truth disease stage labels $y_{j}$ (with optional spatial masks $p_{j}$). The outputs at this stage are the predicted disease stage probabilities ${\hat{y}}_{j}$ and auxiliary spatial predictions ${\hat{p}}_{j},$ which are collectively used to compute the joint loss for end-to-end optimization. Conversely, during the inference stage, the model requires only the real-time aligned multimodal data streams ({I*_j_*} and $\left\{{\tilde{e}}_{j}\right\}$) as inputs. The final output is the predicted disease stage probability ${\hat{y}}_{j}$ and the corresponding localized risk regions, which serve as direct, actionable early warning signals for greenhouse management.

#### 3.3.2 Environment-guided visual attention module

**Figure 3:**
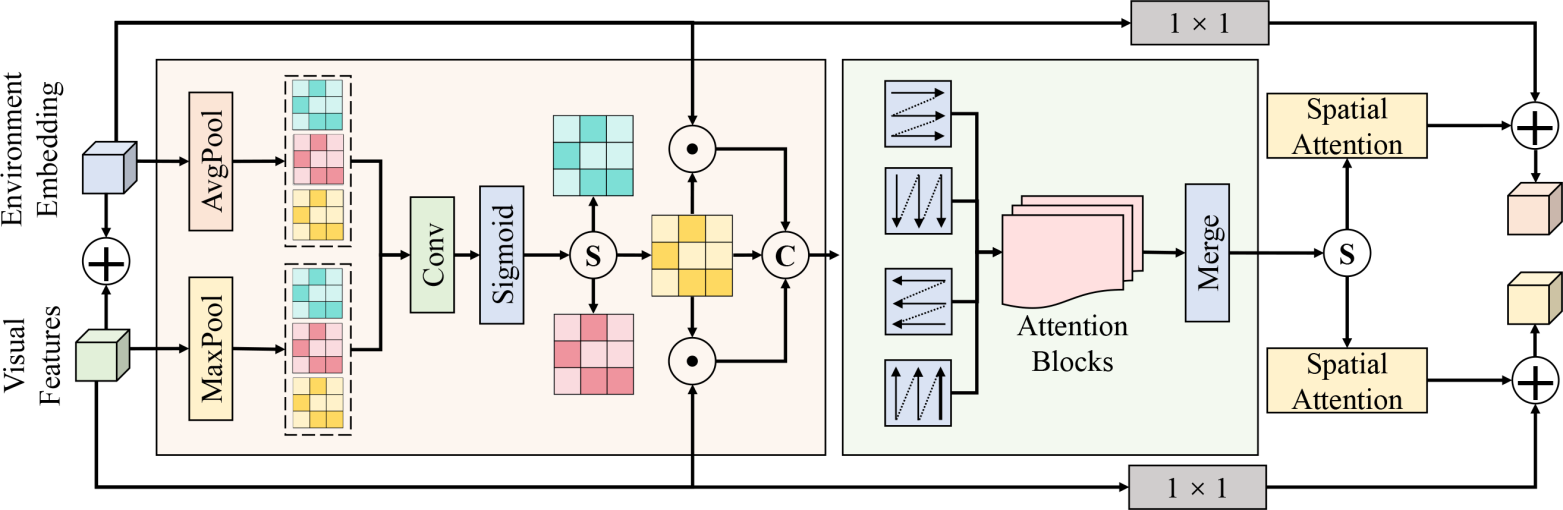
Detailed illustration of the environment-guided visual attention module. The module aligns the Environment Embedding with Visual Features to generate conditional attention weights. Visual features are spatially aggregated via parallel Average Pooling (AvgPool) and Maximum Pooling (MaxPool) layers, followed by convolution and Sigmoid activation. Simultaneously, the environment embedding is transformed to modulate these visual responses through element-wise multiplication (⊙) and concatenation (C). The modulated representations are further refined by stacked Attention Blocks and Spatial Attention mechanisms, before being integrated with the original visual features via 1 × 1 convolutional residual connections to enhance responses to potential disease regions.

The environment-guided visual attention module is designed to address the inherent imbalance in early disease scenarios, where visual cues are weak while environmental priors are strong. Unlike conventional self-attention mechanisms that rely solely on modeling correlations within homogeneous visual features, the proposed module explicitly introduces environmental semantics as external guidance signals to directionally modulate spatial responses of visual features. In classical self-attention, attention weights are generated exclusively from visual features by measuring similarities among queries, keys, and values, which essentially constitutes an autocorrelation modeling process. In contrast, environment-guided attention conditions the generation of attention weights on the temporal environmental representation produced by the sensor branch, thereby realizing cross-modal conditional attention. This design allows potential risk regions to be emphasized in advance according to environmental trends even when clear lesion structures have not yet formed, preventing self-attention from degenerating into noise amplification under weak-texture conditions. Whereas self-attention emphasizes internal visual consistency, environment-guided attention focuses on causal associations between environment and vision, aiming not to reconstruct global visual relations but to introduce biased guidance and suppression in visual responses. As shown in **Figure 3**, this module takes the visual feature map $F\in {\mathbb{R}}^{H\times W\times C}$ from a given backbone stage as input, where *H* × *W* denotes spatial resolution and *C* the number of channels, while simultaneously receiving the environmental embedding on the sensor branch at the same temporal step. Global average pooling and global max pooling are first applied to F along the spatial dimension, yielding two channel descriptors $f_{\text{avg}},f_{\text{max}}\in {\mathbb{R}}^{C}$. After concatenation, a 1 × 1 convolution and nonlinear mapping are used to generate the initial visual channel response. The environmental embedding **s** is mapped through a two-layer fully connected network to a channel-aligned environmental condition vector $g\in {\mathbb{R}}^{C}$. Channel-wise modulation is then performed via element-wise multiplication, which can be expressed as Equations 5, 6.

(5) $$a_{c}=\sigma (W_{c}[f_{\text{avg}};f_{\text{max}}]),\;m_{c}=\sigma (W_{e}s),$$

(6) $${\tilde{F}}_{c}(x,y)=F_{c}(x,y)\cdot a_{c}\cdot m_{c},$$

where *σ*(·) denotes the Sigmoid function, and *W_c_* and *W_e_* are learnable parameters for channel attention and environmental mapping, respectively. Specifically, the visual channel attention factor **a***_c_* evaluates the intrinsic visual information to highlight channels capturing subtle structural or textural anomalies (e.g., slight leaf chlorosis). Concurrently, the environmental regulatory factor **m***_c_* acts as an active risk prior, selectively amplifying the specific visual channels that are strongly correlated with the prevailing environmental disease-inducing conditions (e.g., high humidity or abnormal temperature fluctuations). Through their joint multiplication, the visual features are dynamically re-weighted: even if the morphological symptoms are extremely weak, their corresponding feature responses are significantly boosted when the environmental data indicates a high risk of disease incubation. This synergistically guided representation enables the subsequent spatial attention submodule (based on a 1 × 1 convolution to generate $A_{\text{s}}\in {\mathbb{R}}^{H\times W}$) to accurately localize and focus on potential disease areas before distinct lesions fully emerge. The output is then injected back into the main feature stream through residual connections.

From a probabilistic perspective, this module can be interpreted as an approximation of the conditional distribution p(F|s). Conventional self-attention implicitly assumes that p(F) can be sufficiently characterized by internal visual structure, an assumption that often fails in early disease scenarios. By introducing the environmental condition s, attention weights are jointly determined by a*_c_* and m*_c_*, effectively incorporating a prior constraint during optimization and enforcing environment-consistent feature learning. In conjunction with the subsequent spatial–temporal attention module, the environment-guided output $\tilde{F}$ serves as a cleaner input for temporal modeling, allowing long-term dependencies to be learned on noise-suppressed spatial features and preventing cumulative amplification of environmental noise over time. This module thus acts as a critical bridge for cross-modal collaboration, complementing spatial attention for localization and temporal attention for evolution modeling, and jointly supporting stable perception of weak early-stage disease features.

#### 3.3.3 Spatial–temporal joint attention for disease evolution modeling

The spatial–temporal joint attention module is built upon the outputs of the environment-guided visual attention module and aims to simultaneously characterize local spatial diffusion patterns and gradual temporal evolution of disease progression within a unified framework.

**Figure 4:**
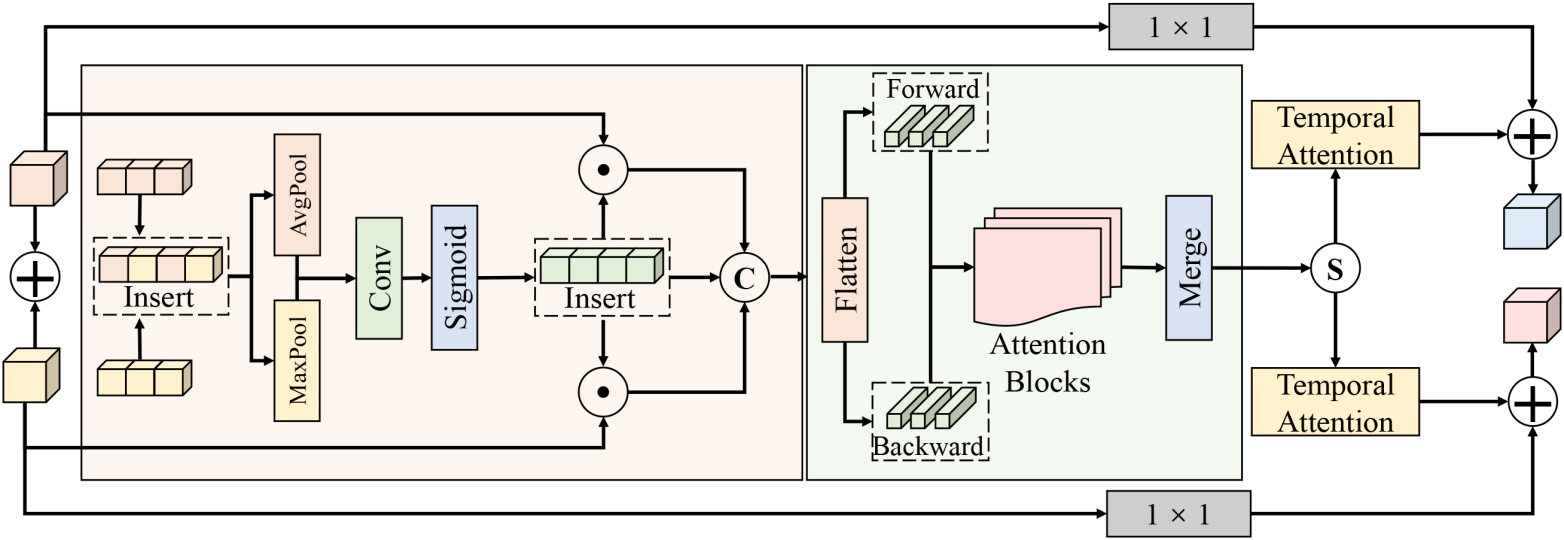
Illustration of the spatial–temporal joint attention module for disease evolution modeling. The process begins with spatial saliency enhancement, where input features undergo pooling (AvgPool/MaxPool) and convolutional operations to highlight potential lesion regions. The spatially enhanced features are then flattened and fed into a bidirectional temporal modeling block consisting of parallel Forward and Backward attention blocks. This structure captures the historical accumulation and progression of the disease. The temporal features are subsequently merged, processed through Temporal Attention layers, and combined with the input features via 1 × 1 convolutional residual connections to produce the final comprehensive representation.

As shown in **Figure 4**, the environment-modulated visual feature sequence is denoted as ${\left\{{\tilde{F}}_{t}\right\}}_{t=1}^{T}$, where ${\tilde{F}}_{t}\in {\mathbb{R}}^{H\times W\times C}$. Within the adopted multi-scale backbone, the feature resolutions correspond to (*H/*8*, W/*8, 96), (*H/*16*, W/*16, 192), and (*H/*32*, W/*32, 384) at different stages. Spatial modeling is first applied to each temporal slice to capture potential lesion distributions and diffusion regions. Specifically, channel-wise max pooling and average pooling are performed on ${\tilde{F}}_{t}$ to obtain $f_{t}^{\text{max}},f_{t}^{\text{avg}}\in {\mathbb{R}}^{H\times W}$. After concatenation, a 3 × 3 convolution and nonlinear mapping generate a spatial weight map $A_{t}\in {\mathbb{R}}^{H\times W}$, which is used to reweight the original features and yield spatially enhanced representations **S***_t_*. This process can be formulated as Equation 7.

(7) $$A_{t}=\sigma (\phi ([f_{t}^{\text{avg}};f_{t}^{\text{max}}])),\;S_{t}(x,y,c)={\tilde{F}}_{t}(x,y,c)\cdot A_{t}(x,y),$$

where *ϕ*(·) denotes convolutional mapping and *σ*(·) the Sigmoid function. The spatial attention mechanism strengthens responses to local weak anomalies during early disease stages while maintaining spatial consistency across time steps, thereby providing a stable basis for temporal modeling.

After spatial enhancement, joint modeling is performed along the temporal dimension. Each S*_t_* is flattened into a sequence of *HW* tokens and linearly projected into a *d* = 256 dimensional embedding space, resulting in $z_{t}\in {\mathbb{R}}^{HW\times d}$. A bidirectional temporal modeling structure is then introduced to capture both forward disease development and backward attribution of key environmental stages. Forward and backward sequences are separately processed by encoders composed of six stacked temporal attention blocks, each including multi-head temporal attention and a feed-forward network. The number of attention heads is set to 8, and the hidden dimension of the feed-forward layers is set to 4*d*. The forward and backward outputs are concatenated and fused along the temporal dimension to obtain the joint temporal representation $H\in {\mathbb{R}}^{T\times d}$. The core temporal attention computation is given by Equations 8, 9.

(8) $$Q_{t}=W_{Q}z_{t},\;K_{\tau }=W_{K}z_{\tau },\;V_{\tau }=W_{V}z_{\tau },$$

(9) $$h_{t}=\sum_{\tau =1}^{T}\frac{\text{exp}(Q_{t}K_{\tau }^{⊤}/\sqrt{d})}{\sum_{k=1}^{T}\text{exp}(Q_{t}K_{\tau }^{⊤}/\sqrt{d})}V_{\tau },$$

where W*_Q_*, W*_K_*, and W*_V_* are learnable projection matrices. This global temporal weighting mechanism adaptively selects the most informative historical stages without relying on fixed temporal windows, enabling effective modeling of long-term cumulative environmental effects on disease evolution.

From a theoretical standpoint, spatial–temporal joint attention corresponds to an optimal weighted estimation of feature sequences under the conditional probability *p*(disease*_t_*|S_1:_*_t_*). Spatial attention approximates local saliency modeling of *p*(S*_t_*|disease*_t_*), while temporal attention approximates the optimal historical weight distribution that minimizes predictive uncertainty. Since attention weights are normalized, the resulting outputs lie within a convex combination space, ensuring numerical stability during training. The module is tightly coupled with the preceding environment-guided visual attention: the latter suppresses spatial noise and highlights potential lesion regions within individual time steps, while the former models diffusion trajectories and evolutionary rhythms over time. In the greenhouse disease early detection task addressed in this work, such a design enables simultaneous capture of where lesions originate and under which environmental stages significant changes occur, allowing stable and interpretable predictions even before visible symptoms fully develop.

#### 3.3.4 Joint loss function

The joint loss function is designed to simultaneously constrain discriminative correctness, early-stage sensitivity, and cross-modal consistency, which fundamentally differs from traditional loss functions that optimize a single objective, such as classification cross-entropy or pixel-wise segmentation loss. Conventional losses implicitly assume equal reliability of supervision across all samples and time points and typically consider either a single modality or a fully fused representation. In the present task, however, early-stage disease samples inherently exhibit weaker visual evidence, higher noise, and stronger dependence on environmental priors. Under a single supervision term, the optimization process tends to bias toward strong features from symptomatic stages, leading to degraded early recall and allowing the environmental branch to act as an unconstrained noise source. To address this issue, a joint loss is constructed that integrates main task supervision, early-stage weighted constraints, and environment–vision alignment regularization into a unified objective, enabling gradients to jointly act on the prediction head, attention modulation, and temporal fusion modules, thereby forming an end-to-end collaborative learning loop.

As shown in **Algorithm 1**, let the predicted disease stage probability at temporal index *j* be ${\hat{y}}_{j}\in {\mathbb{R}}^{C}\;$with ground truth y*_j_*, and let optional pixel- or region-level predictions be denoted by ${\hat{p}}_{j}$ and p*_j_*. The environmental semantic vector produced by the sensor branch at time *j* is $z_{j}^{\text{env}}\in {\mathbb{R}}^{D},$ while the globally pooled visual representation after environment-guided attention is $z_{j}^{\text{vis}}\in {\mathbb{R}}^{D}$. The overall loss is defined as Equation 10.

(10) $$L=L_{\text{sup}}+\lambda_{\text{early}}L_{\text{early}}+\lambda_{\text{align}}L_{\text{align}}+\lambda_{\text{ds}}L_{\text{ds}}.$$

Algorithm 1

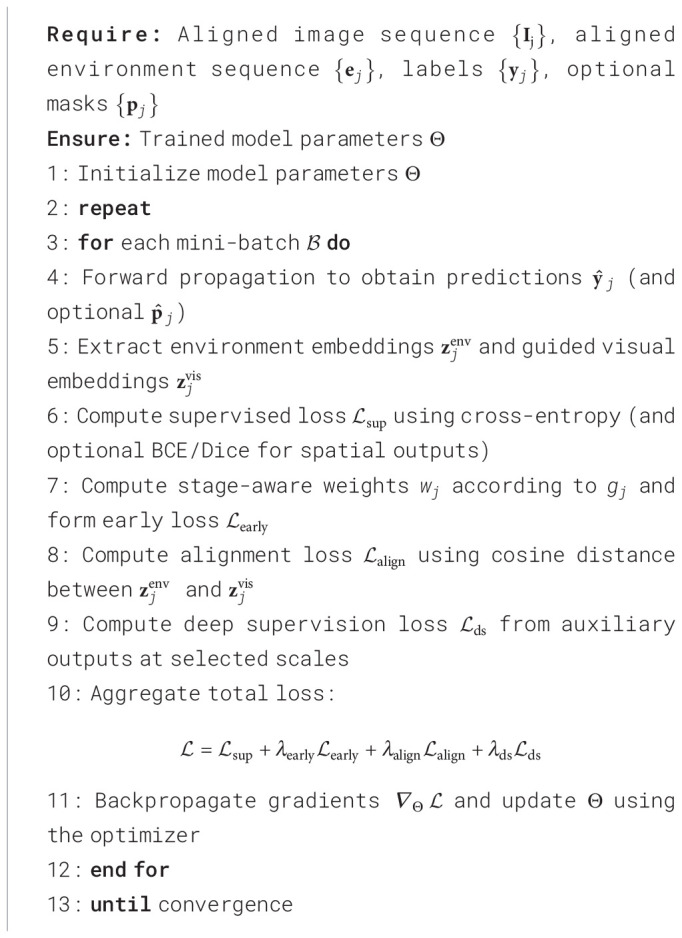

In our experiments, to balance the contributions of different objectives, the weight coefficients are empirically set to *λ*_early_ = 0.5, *λ*_align_ = 0.1, and *λ*_ds_ = 0.3. The main supervision term $L_{\text{sup}}$, as Equation 11, adopts cross-entropy for stage classification, optionally combined with Dice or BCE loss for spatial outputs to enhance learning on small lesions:

(11) $$L_{\text{sup}}=-\frac{1}{M}\sum_{j=1}^{M}\sum_{c=1}^{C}y_{j,c}\log\;{\hat{y}}_{j,c}+\beta \text{BCE}({\hat{p}}_{j},p_{j}).$$

The early-stage constraint, as Equation 12, explicitly assigns higher weights to early samples or early temporal segments, suppressing solutions that are correct only at symptomatic stages:

(12) $$L_{\text{early}}=-\frac{1}{M}\sum_{j=1}^{M}w_{j}\sum_{c=1}^{C}y_{j,c}\log\;{\hat{y}}_{j,c},\;w_{j}=1+\gamma \;I(g_{j}=\text{early}),$$

where *g_j_* denotes the disease stage label and I(·) is the indicator function. The cross-modal alignment term, as Equation 13, constrains environmental guidance to be supportive rather than disruptive by enforcing directional consistency between $z_{j}^{\text{env}}$ and $z_{j}^{\text{vis}}$ in a shared semantic space:

(13) $$L_{\text{align}}=\frac{1}{M}\sum_{j=1}^{M}(1-\frac{\langle z_{j}^{\text{env}},z_{j}^{\text{vis}}\rangle }{\|z_{j}^{\text{env}}{\|}_{2}\|z_{j}^{\text{vis}}{\|}_{2}+\epsilon }).$$

The deep supervision term $L_{\text{ds}}$, as Equation 14, incorporates auxiliary outputs from intermediate scales to mitigate gradient attenuation caused by multi-level attention and temporal modeling, enabling early weak features to receive effective learning signals:

(14) $$L_{\text{ds}}=\sum_{\ell \in \text{Ω}}\alpha_{\ell }\;L_{\text{sup}}^{\left(\ell \right)},$$

where Ω denotes the set of supervised scales. From an optimization perspective, when *λ*_early_*, λ*_align_*, λ*_ds_ ≥ 0, the joint objective is a non-negative linear combination of bounded terms and thus admits a lower bound. Moreover, $L_{\text{align}}$ provides a stable gradient path from environmental semantics to attention weights and fusion layers under weak visual evidence, improving early-stage learnability and reducing overfitting to incidental texture noise. The early-weighted term effectively performs risk reweighting across disease stages, aligning empirical risk minimization with the application utility of early warning. Consequently, in the greenhouse disease detection task, the proposed joint loss typically yields higher early recall, stronger cross-season generalization, and more interpretable attention patterns.

## 4 Results and discussion

### 4.1 Experimental configuration

#### 4.1.1 Hardware and software platform

All experiments were conducted on a dedicated deep learning server running the Ubuntu 22.04 LTS operating system. The hardware configuration included an Intel Xeon Gold 6248R CPU, 256 GB of system RAM, and two NVIDIA GeForce RTX 3090 GPUs (24 GB VRAM each). This robust configuration ensured sufficient memory capacity and parallel computing power to handle the extensive caching of large-scale multimodal temporal data and the end-to-end training of the complex dual-branch deep network. On the software side, the experimental framework was implemented using Python 3.9 and the PyTorch 2.0.1 deep learning library, accelerated by CUDA 11.8 and cuDNN 8.7. Consistent dependency versions were strictly maintained throughout the study to avoid discrepancies caused by software heterogeneity and to guarantee the exact reproducibility of all experimental results.

To strictly prevent temporal data leakage, the dataset was partitioned based on independent disease incidence events and distinct crop planting cycles, rather than random frame-level or sample-level shuffling. Specifically, all continuous multimodal temporal sequences belonging to the same plant or the same localized infection event were strictly isolated and assigned in their entirety to either the training, validation, or testing set. Under this event-based isolation constraint, a 5-fold cross-validation strategy was employed. In each fold, the independent subsets were distributed for training, validation (for hyperparameter tuning), and testing, and the final performance was reported as the average across the 5 folds, thereby ensuring an unbiased assessment of the model’s genuine early warning capability in unseen scenarios.

Key hyperparameters were systematically determined through a combination of grid search on the validation set and domain-specific agronomic prior knowledge. The network was optimized using the AdamW optimizer with a weight decay of 1 × 10^−4^ to mitigate overfitting. The initial learning rate was set to 1 × 10^−4^ and was gradually adjusted using a cosine annealing decay schedule to stabilize late-stage convergence. The training batch size was set to 16, carefully balancing the GPU memory limits with the stability of multimodal gradient estimation. For the environmental preprocessing module, as discussed, the temporal window half-length (Δ*t*) was explicitly set to 30 minutes to capture hourly microclimate phase transitions, and the exponential smoothing coefficient (*α*) was set to 0.2. In the temporal modeling module, the attention embedding dimension was fixed at 256. All network parameters were initialized using the Xavier uniform distribution. This standardized hyperparameter configuration was uniformly applied across all cross-validation folds to ensure optimal training efficiency and strong model generalization.

#### 4.1.2 Baseline models and evaluation metrics

A set of representative baseline models was selected to evaluate early disease detection performance from different modeling paradigms. The CNN model based on RGB images, as a classical visual approach Purwono et al. (2022), is capable of efficiently extracting local texture and morphological features and exhibits stable performance in the recognition of symptomatic lesions. By integrating CNN with LSTM Zha et al. (2022), temporal sequence modeling capability is further introduced, enabling the utilization of dynamic variations in consecutive images and enhancing the characterization of disease evolution processes. The Swin Transformer Liu et al. (2022) enhances global spatial modeling capacity through hierarchical window-based self-attention mechanisms, providing stronger feature representation under complex background conditions. Building upon this, the Temporal Transformer Geng et al. (2022) performs global temporal modeling on image sequences, effectively capturing long-range dependencies and making it suitable for describing overall disease progression over time. The CNN+Env Talaat et al. (2022) and Transformer+Env Shao et al. (2022) models incorporate environmental information through multimodal feature fusion, introducing greenhouse environmental factors into the decision process and providing additional prior knowledge for disease identification. The multimodal Transformer Xu et al. (2023) further conducts unified modeling of visual and environmental information within a single framework, enhancing cross-modal feature representation capability. The multimodal attention model without environment guidance Schroer and Yu (2023) strengthens key information aggregation through attention mechanisms and demonstrates solid overall performance in multimodal scenarios. Together, these baseline models form a comprehensive and representative comparison system.

Model performance was systematically evaluated using classification accuracy, recall, F1-score, and a lead-time metric tailored to early disease detection, thereby enabling assessment from multiple perspectives, including overall recognition performance, the risk of missed detections, and early warning capability. Classification accuracy was used to quantify overall discriminative capability across all samples, recall reflected the coverage of true disease samples, and F1-score characterized the balance between precision and recall. The lead-time metric was introduced to quantify how much earlier a warning could be generated relative to manual inspection or the onset of obvious symptoms. The mathematical definitions of these metrics are given as Equations 15–19:

(15) $$\text{Accuracy}=\frac{TP+TN}{TP+TN+FP+FN},$$

(16) $$\text{Recall}=\frac{TP}{TP+FN},$$

(17) $$\text{Precision}=\frac{TP}{TP+FP},$$

(18) $$\text{F}1-\text{score}=2\cdot \frac{\text{Precision}\cdot \text{Recall}}{\text{Precision}+\text{Recall}},$$

(19) $$\text{LeadTime}=\frac{1}{N}\sum_{i=1}^{N}(t_{i}^{\text{det}}-t_{i}^{\text{ref}}),$$

where *TP*, *TN*, *FP*, and *FN* represent the standard components of the confusion matrix. $t_{i}^{\text{det}}$ denotes the first time point the model predicts the *i*-th sample as diseased, $t_{i}^{\text{ref}}$ is the reference time point determined by expert annotation, and *N* is the total number of diseased samples evaluated. To prevent transient noise from triggering false warnings, the determination of $t_{i}^{\text{det}}$ integrates a temporal hysteresis mechanism: it is recorded only when the predicted disease probability exceeds 0.5 for three consecutive observations. Because the lead-time metric is computed exclusively on true positive trajectories, it is rigorously evaluated in conjunction with Precision and F1-score to ensure that extended early warnings are not achieved at the cost of massive false alarms on healthy samples. Methodologically, lead time is strictly an a posteriori evaluation metric computed during the testing phase, rather than a target label or hyperparameter explicitly optimized during training. Its extension naturally emerges from the model’s enhanced sensitivity to early weak symptoms. Finally, because the sliding window for environmental preprocessing operates on a micro-temporal scale (minutes), it neither introduces future data leakage nor compromises the validity of the lead-time calculation, which evaluates disease incubation on a macro-temporal scale (days).

### 4.2 Performance comparison with baseline models

The primary objective of this experiment is to systematically evaluate the overall performance advantages of the proposed method in early greenhouse crop disease detection and to verify the effectiveness and limitations of different modeling paradigms under early disease perception scenarios through comparisons with multiple representative baseline models. The selected baselines cover a wide range of mainstream technical routes, including single-modality visual modeling, visual temporal modeling, simple multimodal fusion, and multimodal attention-based modeling, thereby providing a comprehensive reflection of the current state of related research. By adopting unified data splits and evaluation metrics, the experiment focuses on key indicators such as accuracy, recall, F1-score, and lead time, enabling a joint analysis of discrimination capability, missed detection risk, and early warning value. This comparative design not only facilitates the validation of the proposed method but also provides quantitative evidence for understanding the roles of environmental information, temporal modeling, and attention mechanisms in early disease detection.

**Figure 5:**
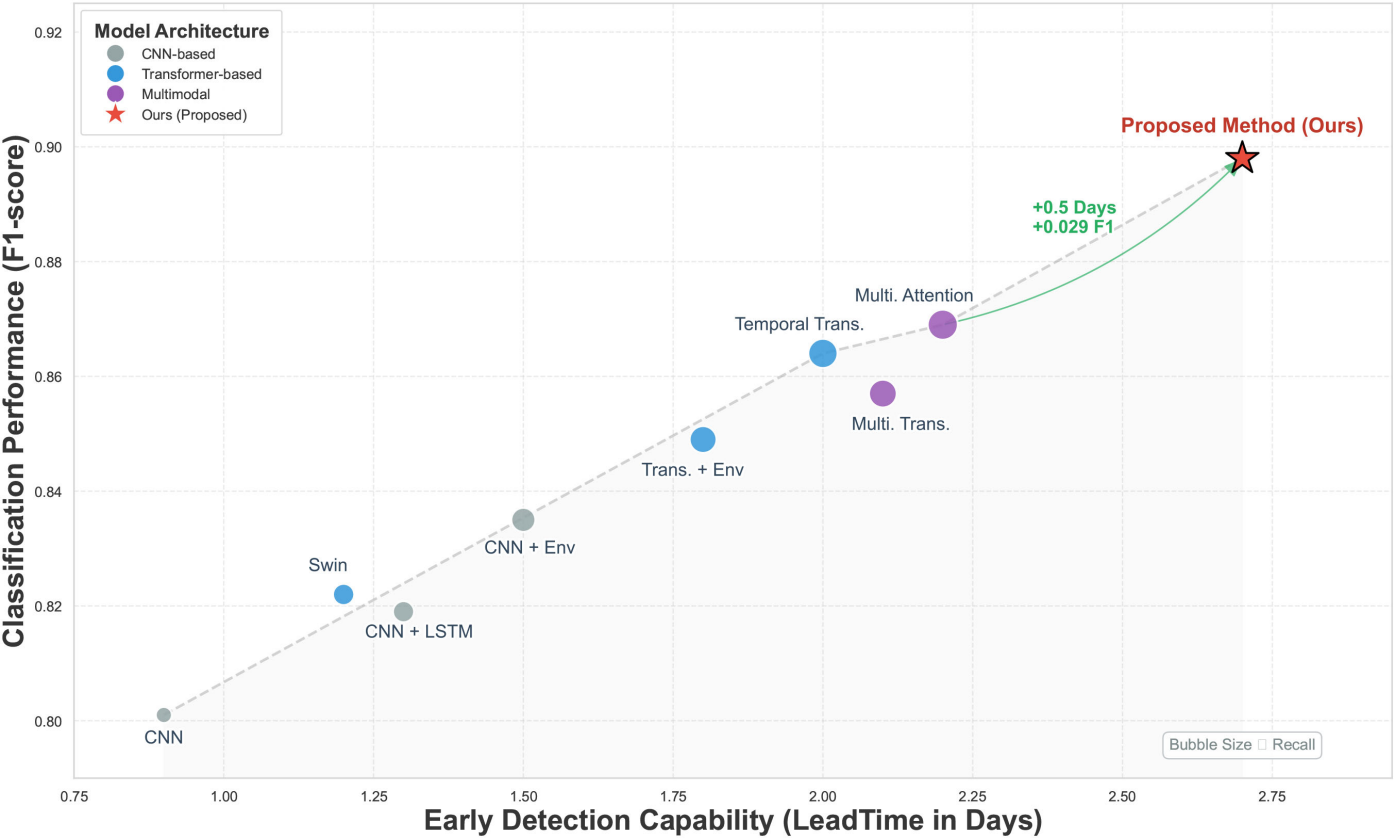
The bubble chart illustrates the trade-off between LeadTime and F1-score across different model architectures. Bubble size is proportional to Recall, indicating the model’s ability to minimize missed detections.

As shown in **Table 2** and **Figure 5**, the CNN model based solely on RGB images exhibits relatively low performance across all metrics, particularly in lead time, indicating that reliance on static visual features alone is insufficient for capturing subtle changes during disease incubation. After incorporating LSTM, the CNN+LSTM model achieves improvements in both recall and lead time, demonstrating that temporal modeling helps exploit dynamic information from image sequences, although its capability remains constrained by the limited long-range dependency modeling of recurrent structures. The Swin Transformer shows a modest overall improvement compared with conventional CNNs, reflecting the advantage of self-attention in spatial modeling; however, without explicit temporal modeling or environmental information, its early detection capability remains limited. The Temporal Transformer, which performs global temporal modeling on image sequences, achieves clear gains in recall and lead time, highlighting the importance of long-term temporal dependencies for disease evolution modeling. With the introduction of environmental information, CNN+Env and Transformer+Env obtain stable performance improvements, yet because feature concatenation or late fusion is adopted, environmental variables mainly act as auxiliary features rather than imposing effective constraints during feature learning. Multimodal Transformer and multimodal attention models without environment guidance further approach optimal performance, suggesting that cross-modal attention enhances feature representation, although attention weights remain predominantly visually driven. In contrast, the proposed method achieves the best performance across all metrics, with a particularly pronounced advantage in lead time. From a modeling perspective, this indicates that environment-guided attention combined with spatial–temporal joint modeling introduces effective prior constraints during feature learning, enabling stable discriminative representations to be formed before disease symptoms become visually apparent, thereby supporting earlier and more reliable detection.

**Table 2:** Performance comparison with baseline models on early disease detection.

Method	Accuracy	Recall	F1-score	LeadTime (days)
CNN (RGB only)	0.842 ± 0.012	0.781 ± 0.015	0.801 ± 0.014	0.9 ± 0.1
CNN + LSTM (RGB)	0.858 ± 0.011	0.803 ± 0.013	0.819 ± 0.012	1.3 ± 0.2
Swin Transformer (RGB only)	0.865 ± 0.010	0.804 ± 0.012	0.822 ± 0.011	1.2 ± 0.1
Temporal Transformer (RGB)	0.891 ± 0.009	0.852 ± 0.010	0.864 ± 0.010	2.0 ± 0.2
CNN + Env (feature concat)	0.872 ± 0.010	0.819 ± 0.012	0.835 ± 0.011	1.5 ± 0.2
Transformer + Env (late fusion)	0.884 ± 0.009	0.836 ± 0.011	0.849 ± 0.010	1.8 ± 0.2
Multimodal Transformer (early fusion)	0.889 ± 0.008	0.842 ± 0.010	0.857 ± 0.009	2.1 ± 0.2
Multimodal Attention (no env guidance)	0.895 ± 0.008	0.858 ± 0.009	0.869 ± 0.009	2.2 ± 0.2
Proposed method	**0.913 ± 0.006**	**0.889 ± 0.008**	**0.898 ± 0.007**	**2.7 ± 0.1**

Bold indicates best results.

### 4.3 Early-stage disease detection performance comparison

This experiment is designed to specifically evaluate the perception capability and early warning value of different models at the early stage of disease development rather than focusing solely on overall classification performance. In early disease detection scenarios, visual symptoms are typically characterized by slight color deviations or weak texture disturbances, resulting in insufficient discriminative information and a high noise ratio. Therefore, models are required not only to identify potential disease risks as early as possible but also to control false alarms while issuing early warnings. Accordingly, early precision, early recall, early F1-score, and lead time are introduced to assess model performance from accuracy, completeness, and temporal perspectives. This comparison enables an intuitive analysis of how different modeling strategies adapt to weak-supervision and low signal-to-noise conditions and reveals the role of environmental information and temporal modeling in early disease perception.

**Figure 6:**
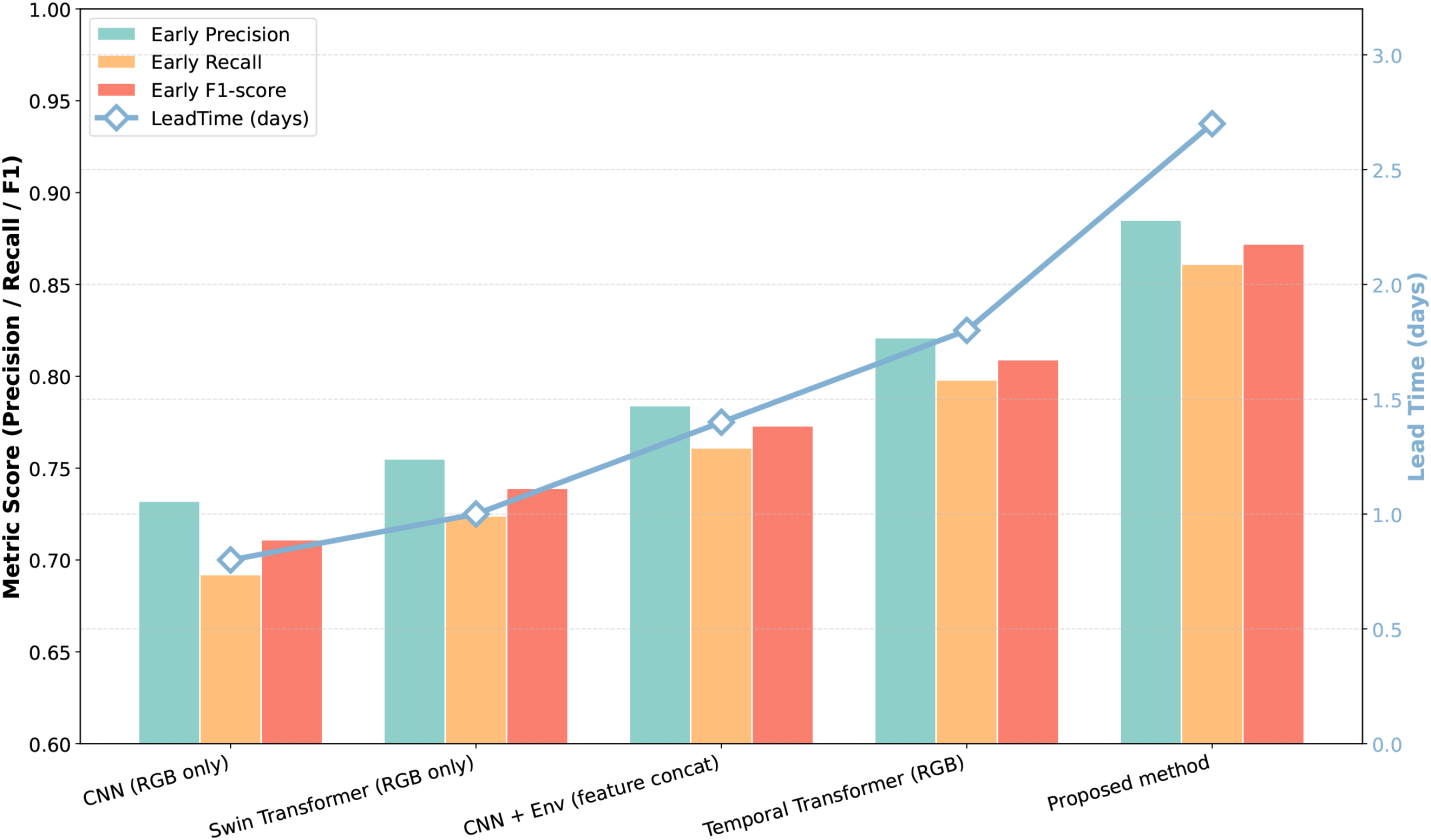
. The dual-axis chart illustrates the model performance across three metrics (Early Precision, Recall, and F1-score) shown as bars on the left axis, and the detection Lead Time (in days) shown as a line on the right axis.

As shown in **Table 3** and **Figure 6**, the CNN based on RGB images alone achieves relatively low early precision and recall, with limited lead time, indicating that its reliance on local spatial textures makes it difficult to distinguish early disease changes from normal physiological variations. The Swin Transformer demonstrates moderate improvements due to its global spatial modeling capability, although its attention weights are still derived solely from visual features and remain susceptible to background interference under weak visual evidence. After environmental information is concatenated with CNN features, early precision and recall are notably improved, confirming that environmental variables contain disease-inducing cues that provide useful prior knowledge for early judgment; however, the guiding role of environmental information remains limited because it does not directly participate in visual feature construction. The Temporal Transformer further improves early-stage performance, reflecting the advantage of long-term temporal modeling in capturing disease incubation trends by accumulating information across multiple time steps. In comparison, the proposed method achieves substantial advantages across all early-stage metrics, especially in lead time. This improvement is theoretically attributable to the environment-guided attention mechanism, which imposes prior constraints on feature distributions and encourages feature aggregation along environment-consistent directions from the early stage, while spatial–temporal joint modeling stably accumulates weak disease signals over time, enabling earlier and more reliable disease warnings.

**Table 3:** Early-stage disease detection performance comparison.

Method	Early precision	Early recall	Early F1-score	LeadTime (days)
CNN (RGB only)	0.732 ± 0.016	0.692 ± 0.018	0.711 ± 0.017	0.8 ± 0.1
Swin Transformer (RGB only)	0.755 ± 0.014	0.724 ± 0.015	0.739 ± 0.014	1.0 ± 0.1
CNN + Env (feature concat)	0.784 ± 0.012	0.761 ± 0.014	0.773 ± 0.013	1.4 ± 0.2
Temporal Transformer (RGB)	0.821 ± 0.011	0.798 ± 0.012	0.809 ± 0.011	1.8 ± 0.2
Proposed method	**0.885 ± 0.007**	**0.861 ± 0.009**	**0.872 ± 0.008**	**2.7 ± 0.1**

Bold indicates best results.

### 4.4 Ablation study of key components

The purpose of this ablation study is to systematically verify the functional contributions and collaborative effects of key components in the proposed framework. By sequentially removing the environment-guided visual attention module, the spatial–temporal joint attention module, the temporal attention mechanism, and the joint loss function while keeping the backbone unchanged, the experiment quantitatively analyzes the independent contributions and coupling effects of different components. Compared with overall performance comparisons, the ablation study emphasizes trends and magnitudes of performance degradation, particularly in lead time, to assess how early disease perception capability deteriorates, thereby verifying whether each component genuinely serves the core objective of early detection. This design also helps exclude the possibility that performance gains arise merely from increased network capacity or parameter count.

**Figure 7:**
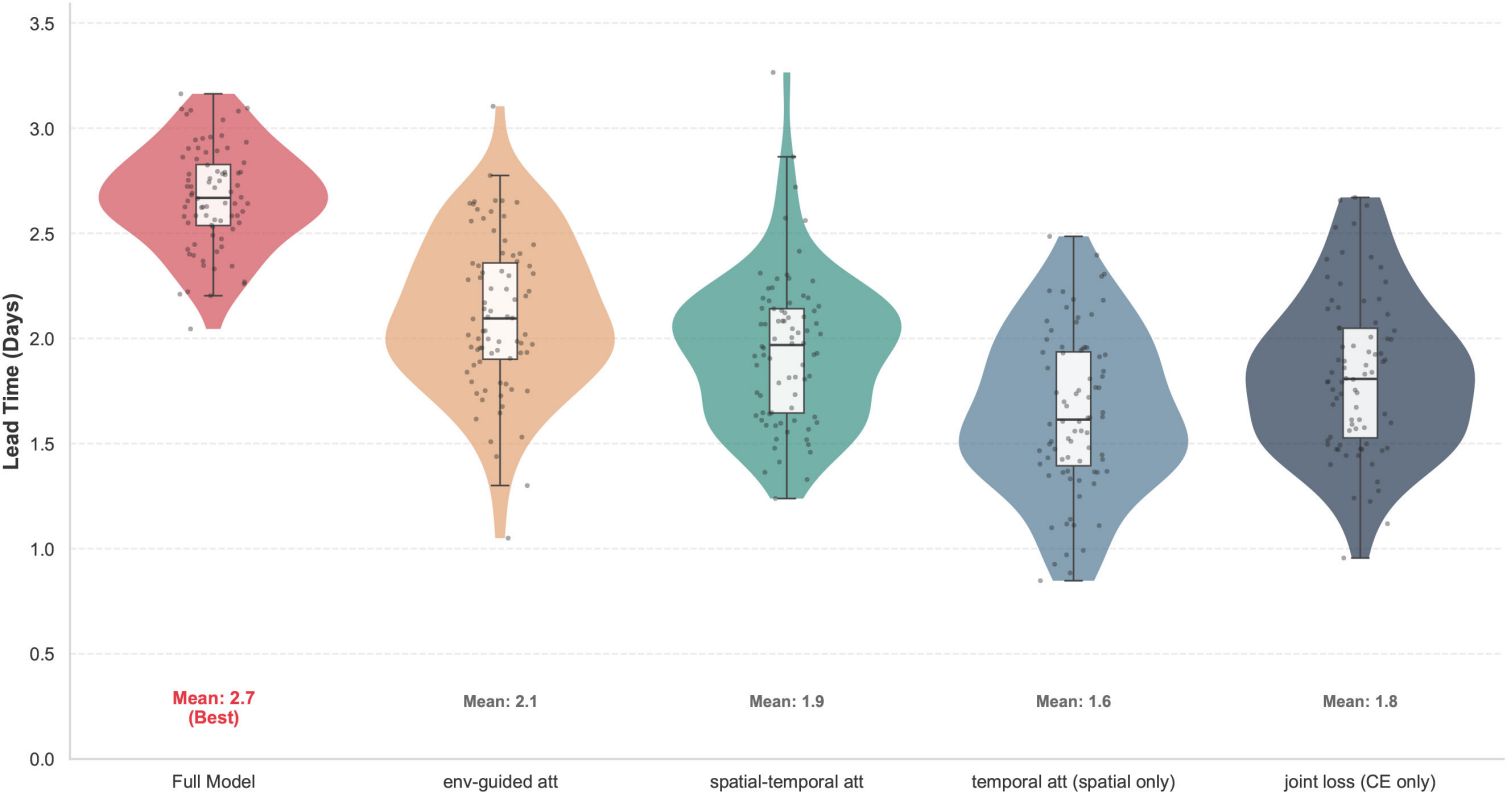
Ablation study on the impact of key components on Lead Time.

As shown in **Table 4** and **Figure 7**, the full model consistently achieves the best performance across all metrics, indicating that the proposed components form a stable and complementary functional collaboration. Removing the environment-guided attention module leads to noticeable drops in accuracy, recall, and especially lead time, suggesting that without constraining visual responses during feature learning, the model becomes more dependent on explicit visual cues and delays disease detection. Eliminating spatial–temporal joint attention further degrades performance, demonstrating that unified modeling of spatial diffusion patterns and temporal evolution is critical for accumulating weak disease signals. When only spatial attention is retained and temporal attention is removed, the lead time decreases most sharply, indicating that single-time-step spatial saliency is insufficient for early warning without cross-time integration. Using only cross-entropy loss also results in performance degradation, implying that without stage sensitivity and cross-modal consistency constraints, optimization tends to bias toward symptomatic-stage samples and weakens early-stage supervision. Overall, the ablation results confirm from both structural and modeling perspectives that environment-guided attention, spatial–temporal joint modeling, and the joint loss function play complementary roles in suppressing noise, stabilizing gradient propagation, and enhancing early signal accumulation, collectively supporting the performance advantages of the proposed framework in early disease detection.

**Table 4:** Ablation study of key components in the proposed framework.

Configuration	Accuracy	Recall	F1-score	LeadTime (days)
Full model	**0.913 ± 0.006**	**0.889 ± 0.008**	**0.898 ± 0.007**	**2.7 ± 0.1**
w/o environment-guided attention	0.894 ± 0.009	0.861 ± 0.010	0.872 ± 0.009	2.1 ± 0.2
w/o spatial–temporal attention	0.887 ± 0.010	0.848 ± 0.012	0.861 ± 0.011	1.9 ± 0.2
w/o temporal attention (spatial only)	0.879 ± 0.011	0.836 ± 0.013	0.849 ± 0.012	1.6 ± 0.2
w/o joint loss (CE only)	0.882 ± 0.010	0.841 ± 0.011	0.855 ± 0.010	1.8 ± 0.2

Bold indicates best results.

### 4.5 Discussion

The results of this study demonstrate clear and direct practical relevance for real-world greenhouse production scenarios. In protected horticulture systems, disease outbreaks are rarely sudden events; instead, they are typically incubated and progressively spread under sustained environmental conditions. Greenhouse managers commonly rely on manual inspection or experiential judgment after visible lesions appear to initiate interventions, which often leads to delayed responses and concentrated or excessive chemical applications. By jointly modeling environmental sensor data and crop images, the proposed approach enables potential risk areas to be identified before lesions become visually evident, by integrating signals such as persistent high humidity, temperature fluctuations, or insufficient ventilation. The achieved improvement in lead time allows localized ventilation adjustment, humidity regulation, or targeted treatment to be conducted prior to disease spread, thereby effectively reducing transmission intensity.

From an operational perspective, the proposed framework is particularly suitable for modern greenhouses in which sensor networks are already widely deployed. In the cultivation of crops such as tomato and cucumber, diseases frequently emerge first in ventilation dead zones, irrigation boundaries, or regions with abnormal microclimates. The environment-guided mechanism introduced in this work emulates the decision logic of experienced growers, in which environmental conditions are assessed prior to symptom inspection. When environmental indicators consistently suggest elevated risk, the system automatically increases attention to corresponding plant regions. This environment-prior visual perception paradigm helps reduce misclassification caused by illumination variation, leaf occlusion, or natural senescence, thereby improving system robustness and interpretability in practical settings. Furthermore, spatial–temporal joint modeling enables perception of disease diffusion trends, allowing warnings to extend beyond individual plants and providing managers with continuous, time-resolved risk evolution information.

At the level of production management, the output format of the proposed method aligns closely with operational requirements. Early warning results can be directly integrated with greenhouse control systems to guide ventilation window operation, dehumidification scheduling, or zone-specific irrigation adjustments, while also providing prioritized regions for manual inspection. This integration significantly reduces inspection workload and dependence on individual experience. Compared with conventional threshold-based warning systems, the proposed method jointly considers the synergistic effects of multiple environmental factors and crop state variations, thereby avoiding frequent false alarms triggered by single-variable fluctuations. In the context of the ongoing transition toward intelligent and fine-grained management in protected agriculture, the proposed visual–environmental disease perception framework provides a technically feasible pathway for achieving early detection, minimal intervention, and precise control.

### 4.6 Limitation and future work

Although the proposed visual–environmental early disease perception method exhibits strong performance and stability in experimental evaluations, several aspects remain open for further investigation and improvement. First, the current framework relies primarily on environmental sensors and fixed-view imaging systems deployed within greenhouses, and its performance may be influenced by sensor density and image coverage. Second, the study focuses on common horticultural crop diseases, and although stable performance is observed across multiple disease types and growth stages, the discriminative capability for rare diseases or compound stress scenarios requires additional validation.

Future research may explore both model adaptability and application expansion. On the one hand, adaptive or continual learning mechanisms could be introduced to allow the model to progressively adjust to long-term changes in greenhouse environments and management strategies, thereby reducing the cost of repeated annotation and centralized retraining. On the other hand, additional information sources, such as crop physiological sensing data, historical management records, or meteorological forecasts, may be integrated to further enhance the modeling of disease-inducing mechanisms.

## 5 Conclusion

Against the background of the continuous transition of protected horticultural production toward large-scale and refined management, achieving reliable early warning before diseases become visually apparent remains a critical practical challenge for ensuring crop yield and quality while reducing chemical inputs. In response to the concealed incubation of diseases in greenhouse environments, the weakness of early visual symptoms, and the strong dependence on environmental conditions, a visual–environment joint early disease perception framework is developed from the perspective of environment-prior-guided visual perception. This framework provides a new technical paradigm for intelligent disease early warning in greenhouses by overcoming the limitations of traditional single-vision approaches or simple multimodal fusion schemes. Environmental information is elevated from passive auxiliary features to active guiding factors, enabling more stable and reliable discrimination during early disease stages.

At the methodological level, an environment-guided visual attention mechanism and a spatial–temporal joint attention modeling strategy are introduced. By imposing environment-consistency constraints during feature learning and accumulating weak disease signals along the temporal dimension, sensitivity to early-stage diseases is effectively enhanced. In addition, a joint loss function tailored for early warning tasks is designed to constrain the model from multiple perspectives, including discrimination correctness, stage sensitivity, and cross-modal consistency, thereby improving training stability and task specificity. Extensive experimental validation on a real-world greenhouse multimodal dataset demonstrates clear advantages of the proposed approach. Compared with multiple representative baseline models, an accuracy of approximately 91.3%, a recall of 88.9%, and an F1-score of 89.8% are achieved in overall detection tasks. In early-stage disease detection scenarios, both early precision and early recall are substantially improved, with the lead time extended to 2.7 days, significantly outperforming methods relying solely on visual information or simple environmental fusion. Ablation studies further confirm the independent contributions and synergistic effects of the key components in improving lead time and maintaining stable detection performance.

Despite these promising results, this study has several limitations that warrant future investigation. The current framework relies primarily on existing sensor networks and fixed-view imaging systems, meaning its performance may be influenced by sensor density and image coverage limits. Furthermore, while the method demonstrates stable performance across common horticultural crop diseases, its discriminative capability for rare diseases or compound stress scenarios requires additional validation. Future research will explore adaptive or continual learning mechanisms to enable the model to progressively adjust to long-term changes in greenhouse environments and management strategies, thereby reducing the cost of centralized retraining. Additionally, integrating supplementary information sources, such as crop physiological sensing data, historical management records, and meteorological forecasts, will be investigated to further enhance the modeling of disease-inducing mechanisms and broaden the application scope of the early warning system.

## Data Availability

The raw data supporting the conclusions of this article will be made available by the authors, without undue reservation.
